# Supplementary material for: Effects of internet-based telemonitoring platforms on the quality of life of oncologic patients: A systematic literature review protocol
Source: PLoS One. 2023 Nov 8;18(11):e0293948. doi: 10.1371/journal.pone.0293948 (PMC10631686; doi:10.1371/journal.pone.0293948)
Supplement: S1 Appendix — (DOCX) [file pone.0293948.s001.docx]

**Appendix**

**Search Strategy**

**OVID Medline Epub Ahead of Print, In-Process & Other Non-Indexed Citations, Ovid MEDLINE(R) Daily and Ovid MEDLINE(R) 1946 to Present**

1 exp Neoplasms/ 3813073

2 (cancer$ or neoplas$ or tumo$ or carcinoma$ or hodgkin$ or nonhodgkin$ or adenocarcinoma$ or leuk?emia$ or metasta$ or malignan$ or lymphoma$ or sarcoma$ or melanoma$ or myeloma$ or oncolog$).tw. 4330782

3 1 or 2 5130395

4 exp Telemedicine/ 43889

5 exp Videoconferencing/ 2720

6 Remote Consultation/ 5680

7 (tele-medicine or telemedicine).tw. 18011

8 (tele-monitoring or telemonitoring).tw. 2211

9 (mhealth$ or m-health$ or m?health).tw. 5918

10 (ehealth$ or e-health$ or e?health$).tw. 6983

11 (emedicine$ or e-medicine$ or e?medi$).tw. 178

12 (telemetry or telehealth or tele-health or telehomecare or tele-homecare or telecoaching or tele-coaching or videoconferenc$ or video-conferenc$ or videoconsultation or video-consultation or teleconferenc$ or tele-conferenc$ or teleconsultation or tele-consultation or telecare or tele-care).tw. 26222

13 ((telephone$ or phone$ or cellphone or video$ or internet$ or computer$ or modem or web$ or email or remote$ or distance$ or distant) adj5 (monitoring or consult$)).tw. 20220

14 or/4-13 89508

15 3 and 14 6206

16 (Randomized Controlled Trial or Controlled Clinical Trial or Pragmatic Clinical Trial or Clinical Study or Adaptive Clinical Trial or Equivalence Trial).pt. 687172

17 (Clinical Trial or Clinical Trial, Phase I or Clinical Trial, Phase II or Clinical Trial, Phase III or Clinical Trial, Phase IV or Clinical Trial Protocol).pt. 609067

18 Multicenter Study.pt. 332280

19 Clinical Studies as Topic/ 783

20 exp Clinical Trial/ or exp Clinical Trials as Topic/ or Clinical Trial Protocol/ or Clinical Trial Protocols as Topic/ or exp "Clinical Trial (topic)"/ 1268765

21 Multicenter Study/ or Multicenter Studies as Topic/ or "Multicenter Study (topic)"/ 351448

22 Randomization/ 106916

23 Random Allocation/ 106916

24 Double-Blind Method/ 174735

25 Double-Blind Studies/ 174735

26 Single-Blind Method/ 32596

27 Placebos/ 35926

28 Cross-Over Studies/ or Crossover Procedure/ 54880

29 (random* or sham or placebo*).ti,ab,hw,kf. 1788579

30 ((singl* or doubl*) adj (blind* or dumm* or mask*)).ti,ab,hw,kf. 265349

31 ((tripl* or trebl*) adj (blind* or dumm* or mask*)).ti,ab,hw,kf. 1558

32 (control* adj3 (study or studies or trial* or group*)).ti,ab,hw,kf. 1902949

33 (clinical adj3 (study or studies or trial*)).ti,ab,hw,kf. 1417147

34 (Nonrandom* or non random* or non-random* or quasi-random* or quasirandom*).ti,ab,hw,kf. 53736

35 (phase adj3 (study or studies or trial*)).ti,ab,hw,kf. 175733

36 ((crossover or cross-over) adj3 (study or studies or trial*)).ti,ab,hw,kf. 76142

37 ((multicent* or multi-cent*) adj3 (study or studies or trial*)).ti,ab,hw,kf. 403647

38 allocated.ti,ab,hw. 82421

39 ((open label or open-label) adj5 (study or studies or trial*)).ti,ab,hw,kf. 43934

40 ((equivalence or superiority or non-inferiority or noninferiority) adj3 (study or studies or trial*)).ti,ab,hw,kf. 11802

41 (pragmatic study or pragmatic studies).ti,ab,hw,kf. 581

42 ((pragmatic or practical) adj3 trial*).ti,ab,hw,kf. 7541

43 ((quasiexperimental or quasi-experimental) adj3 (study or studies or trial*)).ti,ab,hw,kf. 11842

44 trial.ti,kf. 302498

45 or/16-44 3750722

46 exp animals/ 26266073

47 exp animal experimentation/ 10299

48 exp models animal/ 638509

49 exp animal experiment/ 10299

50 exp vertebrate/ 25527951

51 or/46-50 26268048

52 exp humans/ 21158240

53 51 not 52 5109808

54 45 not 53 3354912

55 15 and 54 1570

56 55 and 2000:2023.(sa_year). 1526

**Embase <1974 to 2023 March 31>**

1 exp neoplasm/

2 exp malignant neoplasm/

3 (cancer$ or neoplas$ or tumo$ or carcinoma$ or hodgkin$ or nonhodgkin$ or adenocarcinoma$ or leuk?emia$ or metasta$ or malignan$ or lymphoma$ or sarcoma$ or melanoma$ or myeloma$ or oncolog$).tw.

4 1 or 3

5 exp telemedicine/

6 exp videoconferencing/

7 teleconsultation/

8 (tele-medicine or telemedicine).tw.

9 (tele-monitoring or telemonitoring).tw.

10 (mhealth$ or m-health$ or m?health).tw.

11 (ehealth$ or e-health$ or e?health$).tw.

12 (emedicine$ or e-medicine$ or e?medi$).tw.

13 (telemetry or telehealth or tele-health or telehomecare or tele-homecare or telecoaching or tele-coaching or videoconferenc$ or video-conferenc$ or videoconsultation or video-consultation or teleconferenc$ or tele-conferenc$ or teleconsultation or tele-consultation or telecare or tele-care).tw.

14 ((telephone$ or phone$ or cellphone or video$ or internet$ or computer$ or modem or web$ or email or remote$ or distance$ or distant) adj5 (monitoring or consult$)).tw.

15 or/5-14

16 4 and 15

17 (Randomized Controlled Trial or Controlled Clinical Trial or Pragmatic Clinical Trial or Clinical Study or Adaptive Clinical Trial or Equivalence Trial).pt.

18 (Clinical Trial or Clinical Trial, Phase I or Clinical Trial, Phase II or Clinical Trial, Phase III or Clinical Trial, Phase IV or Clinical Trial Protocol).pt.

19 Multicenter Study.pt.

20 Clinical Studies as Topic/

21 exp Clinical Trial/ or exp Clinical Trials as Topic/ or Clinical Trial Protocol/ or Clinical Trial Protocols as Topic/ or exp "Clinical Trial (topic)"/

22 Multicenter Study/ or Multicenter Studies as Topic/ or "Multicenter Study (topic)"/

23 Randomization/

24 Random Allocation/

25 Double-Blind Method/

26 Double Blind Procedure/

27 Double-Blind Studies/

28 Single-Blind Method/

29 Single Blind Procedure/

30 Single-Blind Studies/

31 Placebos/

32 Placebo/

33 Control Groups/

34 Control Group/

35 Cross-Over Studies/ or Crossover Procedure/

36 (random* or sham or placebo*).ti,ab,hw,kf.

37 ((singl* or doubl*) adj (blind* or dumm* or mask*)).ti,ab,hw,kf.

38 ((tripl* or trebl*) adj (blind* or dumm* or mask*)).ti,ab,hw,kf.

39 (control* adj3 (study or studies or trial* or group*)).ti,ab,hw,kf.

40 (clinical adj3 (study or studies or trial*)).ti,ab,hw,kf.

41 (Nonrandom* or non random* or non-random* or quasi-random* or quasirandom*).ti,ab,hw,kf. 69518

42 (phase adj3 (study or studies or trial*)).ti,ab,hw,kf.

43 ((crossover or cross-over) adj3 (study or studies or trial*)).ti,ab,hw,kf.

44 ((multicent* or multi-cent*) adj3 (study or studies or trial*)).ti,ab,hw,kf.

45 allocated.ti,ab,hw.

46 ((open label or open-label) adj5 (study or studies or trial*)).ti,ab,hw,kf.

47 ((equivalence or superiority or non-inferiority or noninferiority) adj3 (study or studies or trial*)).ti,ab,hw,kf.

48 (pragmatic study or pragmatic studies).ti,ab,hw,kf.

49 ((pragmatic or practical) adj3 trial*).ti,ab,hw,kf.

50 ((quasiexperimental or quasi-experimental) adj3 (study or studies or trial*)).ti,ab,hw,kf.

51 trial.ti,kf.

52 or/17-51

53 exp animals/

54 exp animal experimentation/

55 exp models animal/

56 exp animal experiment/

57 nonhuman/

58 exp vertebrate/

59 or/53-58

60 exp humans/

61 exp human experiment/

62 or/60-61

63 59 not 62

64 52 not 63

65 16 and 64

66 65 and 2000:2023.(sa_year).

67 limit 66 to (embase or "preprints (unpublished, non-peer reviewed)")

**Cochrane Library (CENTRAL)**

#1 MeSH descriptor: [Neoplasms] explode all trees

#2 (cancer* or neoplas* or tumo* or carcinoma* or hodgkin* or nonhodgkin* or adenocarcinoma* or leuk?emia* or metasta* or malignan* or lymphoma* or sarcoma* or melanoma* or myeloma* or oncolog*):ti,ab,kw

#3 #1 OR #2

#4 MeSH descriptor: [Telemedicine] explode all trees

#5 MeSH descriptor: [Videoconferencing] explode all trees

#6 MeSH descriptor: [Remote Consultation] explode all trees

#7 (tele-medicine or telemedicine):ti,ab,kw

#8 (tele-monitoring or telemonitoring):ti,ab,kw

#9 (mhealth* or m-health* or m?health):ti,ab,kw

#10 (ehealth* or e-health* or e?health*):ti,ab,kw

#11 (emedicine* or e-medicine* or e?medi*):ti,ab,kw

#12 (telemetry or telehealth or tele-health or telehomecare or tele-homecare or telecoaching or tele-coaching or videoconferenc* or video-conferenc* or videoconsultation or video-consultation or teleconferenc* or tele-conferenc* or teleconsultation or tele-consultation or telecare or tele-care):ti,ab,kw 6848

#13 ((telephone* or phone* or cellphone or video* or internet* or computer* or modem or web* or email or remote* or distance* or distant) near/5 (monitoring or consult*)):ti,ab,kw 4895

#14 {OR #4-#13}

#15 #3 AND #14

#16 Trials 2000-2023

**CINAHL**

S1 (MH "Neoplasms+")

S2 (MH "Cancer Patients")

S3 TI ( cancer* or neoplas* or tumo* or carcinoma* or hodgkin* or nonhodgkin* or adenocarcinoma* or leuk#emia* or metasta* or malignan* or lymphoma* or sarcoma* or melanoma* or myeloma* or oncolog* ) OR AB ( cancer* or neoplas* or tumo* or carcinoma* or hodgkin* or nonhodgkin* or adenocarcinoma* or leuk#emia* or metasta* or malignan* or lymphoma* or sarcoma* or melanoma* or myeloma* or oncolog* ) 777,194

S4 S1 OR S2 OR S3 925,301

S5 (MH "Telemedicine+")

S6 (MH "Telehealth+")

S7 (MH "Videoconferencing+")

S8 (MM "Remote Consultation")

S9 TI ( tele-medicine or telemedicine ) OR AB ( tele-medicine or telemedicine )

S10 TI ( tele-monitoring or telemonitoring ) OR AB ( tele-monitoring or telemonitoring )

S11 TI ( mhealth* or m-health* or m#health ) OR AB ( mhealth* or m-health* or m#health ) 2,847

S12 TI ( ehealth* or e-health* or e#health* ) OR AB ( ehealth* or e-health* or e#health* )

S13 TI ( emedicine* or e-medicine* or e#medi* ) OR AB ( emedicine* or e-medicine* or e#medi* ) 261

S14 TI ( telemetry or telehealth or tele-health or telehomecare or tele-homecare or telecoaching or tele-coaching or videoconferenc* or video-conferenc* or videoconsultation or video-consultation or teleconferenc* or tele-conferenc* or teleconsultation or tele-consultation or telecare or tele-care ) OR AB ( telemetry or telehealth or tele-health or telehomecare or tele-homecare or telecoaching or tele-coaching or videoconferenc* or video-conferenc* or videoconsultation or video-consultation or teleconferenc* or tele-conferenc* or teleconsultation or tele-consultation or telecare or tele-care )

S15 TI ( (telephone* or phone* or cellphone or video* or internet* or computer* or modem or web* or email or remote* or distance* or distant) N5 (monitoring or consult*) ) OR AB ( (telephone* or phone* or cellphone or video* or internet* or computer* or modem or web* or email or remote* or distance* or distant) N5 (monitoring or consult*) )

S16 S5 OR S6 OR S7 OR S8 OR S9 OR S10 OR S11 OR S12 OR S13 OR S14 OR S15

S17 S4 AND S16

S18 (((MH "Experimental Studies+") OR (MH "Multicenter Studies") OR (MH "Random Sample+") OR (MH "Placebos") OR (MH "Control (Research)+") OR (MH "Crossover Design") OR ((TI random* OR AB random*) OR (TI sham OR AB sham) OR (TI placebo* OR AB placebo*)) OR (((TI singl* OR AB singl*) OR (TI doubl* OR AB doubl*)) W1 ((TI blind* OR AB blind*) OR (TI dumm* OR AB dumm*) OR (TI mask* OR AB mask*))) OR (((TI tripl* OR AB tripl*) OR (TI trebl* OR AB trebl*)) W1 ((TI blind* OR AB blind*) OR (TI dumm* OR AB dumm*) OR (TI mask* OR AB mask*))) OR ((TI control* OR AB control*) N3 ((TI study OR AB study) OR (TI studies OR AB studies) OR (TI trial* OR AB trial*) OR (TI group* OR AB group*))) OR ((TI clinical OR AB clinical) N3 ((TI study OR AB study) OR (TI studies OR AB studies) OR (TI trial* OR AB trial*))) OR ((TI Nonrandom* OR AB Nonrandom*) OR (TI "non random*" OR AB "non random*") OR (TI "non-random*" OR AB "non-random*") OR (TI "quasi-random*" OR AB "quasi-random*") OR (TI quasirandom* OR AB quasirandom*)) OR ((TI phase OR AB phase) N3 ((TI study OR AB study) OR (TI studies OR AB studies) OR (TI trial* OR AB trial*))) OR (((TI crossover OR AB crossover) OR (TI "cross-over" OR AB "cross-over")) N3 ((TI study OR AB study) OR (TI studies OR AB studies) OR (TI trial* OR AB trial*))) OR (((TI multicent* OR AB multicent*) OR (TI "multi-cent*" OR AB "multi-cent*")) N3 ((TI study OR AB study) OR (TI studies OR AB studies) OR (TI trial* OR AB trial*))) OR (TI allocated OR AB allocated) OR (((TI "open label" OR AB "open label") OR (TI "open-label" OR AB "open-label")) N5 ((TI study OR AB study) OR (TI studies OR AB studies) OR (TI trial* OR AB trial*))) OR (((TI equivalence OR AB equivalence) OR (TI superiority OR AB superiority) OR (TI "non-inferiority" OR AB "non-inferiority") OR (TI noninferiority OR AB noninferiority)) N3 ((TI study OR AB study) OR (TI studies OR AB studies) OR (TI trial* OR AB trial*))) OR ((TI "pragmatic study" OR AB "pragmatic study") OR (TI "pragmatic studies" OR AB "pragmatic studies")) OR (((TI pragmatic OR AB pragmatic) OR (TI practical OR AB practical)) N3 (TI trial* OR AB trial*)) OR (((TI quasiexperimental OR AB quasiexperimental) OR (TI "quasi-experimental" OR AB "quasi-experimental")) N3 ((TI study OR AB study) OR (TI studies OR AB studies) OR (TI trial* OR AB trial*))) OR TI trial)

S19 S17 AND S18

S20 S17 AND S18 Limiters - Published Date: 20000101-20231231q

**Epistemonikos**

1 (advanced_title_en:(Neoplasm* OR Cancer*) OR advanced_abstract_en:(Neoplasm* OR Cancer*)) AND (advanced_title_en:(Telemedicine OR Tele-medicine OR Telemonitoring OR Tele-monitoring OR Videoconferenc* OR Video-conference OR "remote consultation" OR "remote medicine" OR ehealth OR e-health) OR advanced_abstract_en:(Telemedicine OR Tele-medicine OR Telemonitoring OR Tele-monitoring OR Videoconferenc* OR Video-conference OR "remote consultation" OR "remote medicine" OR ehealth OR e-health)) [Filters: classification=primary-study, protocol=no]

**LILACS**

1 (neoplasm* OR cancer*) AND (telemedicine OR tele-medicine OR telemonitoring OR tele-monitoring OR videoconferenc* OR video-conference OR "remote consultation" OR "remote medicine" OR ehealth OR e-health) AND ( db:("LILACS") AND type_of_study:("clinical_trials")) AND (year_cluster:[2020 TO 2023])

**ClinicalsTrials.gov**

1. Telemedicine OR Tele-medicine OR Videoconferencing OR Remote OR Tele-monitoring OR Telemonitoring | Interventional Studies | Cancer
