# Supplementary material for: Effects of internet-based telemonitoring platforms on the quality of life of oncologic patients: A systematic literature review protocol
Source: PLoS One. 2023 Nov 8;18(11):e0293948. doi: 10.1371/journal.pone.0293948 (PMC10631686; doi:10.1371/journal.pone.0293948)
Supplement: Attachment — Submitted filename: Response Letter.docx [file pone.0293948.s003.docx]

Viña del Mar, Tuesday 26th September 2023

Dear Dr. Bastani,

Thank you for your interest in our manuscript and for raising observations that undoubtedly help us improve the quality of our work. Below, we will respond to the commentaries raised by the review team, which we have divided into two sections to facilitate reading.

Reviewer #1

We regret not having generated sufficient interest in reviewer #1.

Regarding the comments briefly mentioned in the reviewer’s objection, we must point out that our proposal corresponds to a research protocol, so it is not yet in a position to report results or findings. Furthermore, it should be considered that the absence of language restrictions in the search process is commonly viewed as a strength when conducting highly sensitive search strategies, as established by the Cochrane Collaboration.

In our opinion, it is appropriate to report the results of a search strategy in a results section of a systematic review, which is not yet applicable to this text as it is a review protocol. However, this phase of the study has already been completed, and we are in a position to supplement the protocol with the results of the literature search. This has been added as a PRISMA flowchart to the study protocol.

Reviewer #2

We appreciate Reviewer #2's time in evaluating our manuscript and pointing out areas for improvement in our work. We’ll address the suggestions and comments that the reviewer provided sequentially:

a) The "Discussion" section is generally not included in the abstract of a systematic review protocol.

The discussion section has been removed from the abstract.

b) Stating the statistical data from a specific source, such as Chile's Ministry of Health, might not be directly relevant to the broader research synthesis. The inclusion of global statistics will better align with the broader research synthesis being conducted in a systematic review.

We have updated these figures from the introduction to the statistics provided by the World Health Organization regarding cancer on a global level.

c) To further strengthen the rationale, consider briefly discussing the limitations or challenges of traditional follow-up methods for cancer patients.

A paragraph has been added to the introduction to describe current limitations of traditional follow-up methods.

d) It might be beneficial to explicitly state the target population (adult cancer patients) to provide a more precise understanding.

We have specified that the systematic review aims to assess the impact of telemonitoring among adult patients with cancer on primary objectives and outcomes. The methodology section specifies that anyone over 18 years old will be considered an adult.

e) As AMSTAR-2 is primarily a critical appraisal tool for systematic reviews of healthcare interventions, not a guideline for reporting systematic review protocols. It would be more appropriate to emphasize adherence to reporting guidelines like PRISMA-P when describing the protocol's design and methodology.

We appreciate the reviewer's clarification regarding these instruments. We have updated our reporting tool to PRISMA-P and adjusted the reference accordingly.

f) The "Eligibility criteria" section should be explicitly outlined as a heading, and it's equally important to include exclusion criteria based on the PICO elements to provide a clear and comprehensive understanding of the study's scope.

We have outlined the eligibility criteria section in the manuscript using a subheading. Additionally, we have provided a more detailed specification of the exclusion criteria for the systematic review and added a summary table for improved clarity in the report.

g) Define all outcomes, including the prioritization of main and additional outcomes, and provide a rationale for this prioritization to ensure transparency and clarity in the study protocol.

We have prioritized and clearly defined all study outcomes in a new section under the methodology titled 'Study Outcomes.' Additionally, we have provided the reasons behind the prioritization of outcomes in the protocol, as suggested by the reviewer.

h) A brief overview of what data will be collected and how it will be organized can help readers understand the data extraction process.

A brief introduction has been added regarding the information that will be obtained from each study separately and the purposes these data will serve for the subsequent analysis, to enhance readability of the text.

i) Quality Assessment in Subgroup: Address whether study quality will impact subgroup analysis.

We specified that a subgroup analysis based on the methodological quality of the included studies will be conducted in the statistical analysis section of the systematic review.

j) Briefly define Review Manager (RevMan) for non-experts.

A brief description of RevMan has been added to our analysis strategy section.

k) A systematic review typically does not include a discussion section.

Our discussion section has been removed from the manuscript. We have replaced it with a “Summary” section to conclude the systematic review protocol.

We sincerely thank the reviewers for their time assessing our study protocol and hope that these modifications address their concerns regarding our proposal.

Kind regards,

Felipe Martínez on behalf of the authors
